# Dental pulp stem cells as a promising model to study imprinting diseases

**DOI:** 10.1038/s41368-022-00169-1

**Published:** 2022-04-02

**Authors:** Eloïse Giabicani, Aurélie Pham, Céline Sélénou, Marie-Laure Sobrier, Caroline Andrique, Julie Lesieur, Agnès Linglart, Anne Poliard, Catherine Chaussain, Irène Netchine

**Affiliations:** 1 Université de Paris, URP2496, Laboratoire Pathologies, imagerie et biothérapies oro-faciales, Montrouge, France; 2 INSERM, UMRS_938 Centre de Recherche Saint Antoine, Paris, France; 3 APHP, Hôpital de Bicêtre, Endocrinologie et Diabétologie de l’Enfant, Centre de Référence des Maladies Rares du Métabolisme du Calcium et du Phosphate, Le Kremlin-Bicêtre, France; 4 APHP, Hôpital Bretonneau, Centre de Référence des Maladies Rares du Métabolisme du Calcium et du Phosphate et FHU DDS-net, Paris, France; 5 Sorbonne Université, INSERM, UMRS_938, APHP, Hôpital Armand Trousseau, Explorations Fonctionnelles Endocriniennes, Paris, France

**Keywords:** Molecular medicine, Clinical epigenetics

## Abstract

Parental imprinting is an epigenetic process leading to monoallelic expression of certain genes depending on their parental origin. Imprinting diseases are characterized by growth and metabolic issues starting from birth to adulthood. They are mainly due to methylation defects in imprinting control region that drive the abnormal expression of imprinted genes. We currently lack relevant animal or cellular models to unravel the pathophysiology of growth failure in these diseases. We aimed to characterize the methylation of imprinting regions in dental pulp stem cells and during their differentiation in osteogenic cells (involved in growth regulation) to assess the interest of this cells in modeling imprinting diseases. We collected dental pulp stem cells from five controls and four patients (three with Silver-Russell syndrome and one with Beckwith-Wiedemann syndrome). Methylation analysis of imprinting control regions involved in these syndromes showed a normal profile in controls and the imprinting defect in patients. These results were maintained in dental pulp stem cells cultured under osteogenic conditions. Furthermore, we confirmed the same pattern in six other loci involved in imprinting diseases in humans. We also confirmed monoallelic expression of *H19* (an imprinted gene) in controls and its biallelic expression in one patient. Extensive imprinting control regions methylation analysis shows the strong potential of dental pulp stem cells in modeling imprinting diseases, in which imprinting regions are preserved in culture and during osteogenic differentiation. This will allow to perform in vitro functional and therapeutic tests in cells derived from dental pulp stem cells and generate other cell-types.

## Introduction

**Fig. 1:**
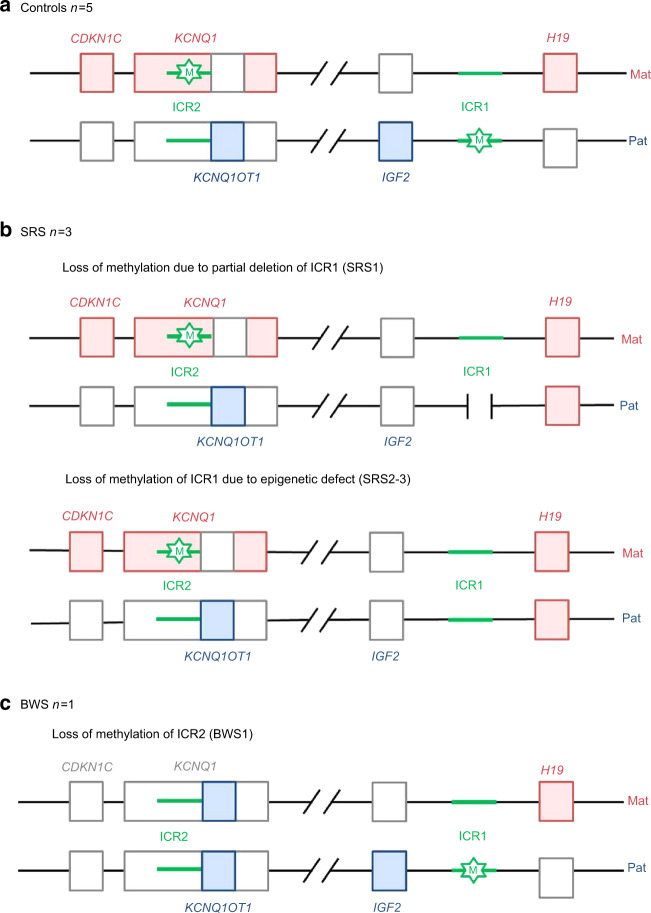
Schematic representation of the imprinted domains in the 11p15 region involved in Silver-Russell and Beckwith-Wiedemann syndromes. Only the imprinted genes that are involved in these syndromes are represented. Blue boxes indicate genes expressed from the paternal (pat) allele (*IGF2* and the long noncoding RNA *KCNQ1OT1*). Red boxes indicate genes expressed from the maternal (mat) allele (*CDKN1c*, *KCNQ1* (coding for an ion channel) and the long noncoding RNA *H19*). Green lines indicate differentially methylated regions (*H19/IGF2*:IG-DMR, the telomeric domain, called ICR1 and *KCNQ1OT1*:TSS-DMR, the centromeric, domain called ICR2). Green stars represent methylated DMRs. **a** Normal 11p15 region. ICR1 is methylated on the paternal allele, leading to *IGF2* expression from the paternal allele and *H19* expression from the maternal allele. ICR2 is methylated on the maternal allele, leading to *CDKN1c* and *KCNQ1* expression from the maternal allele and *KCNQ1OT1* expression from the paternal allele. **b** The three SRS patients (SRS1-3) presented with the loss of methylation of ICR1 in the paternal allele due to partial deletion of ICR1 (SRS1) or an epigenetic defect (SRS2-3), leading to a lack of paternal expression of *IGF2* and gain of maternal expression of *H19*. **c** The BWS patient presented with the loss of methylation of ICR2 in the maternal allele, leading to a lack of maternal expression of *CDKN1c* and a gain of paternal expression of *KCNQ1OT1*

Imprinting diseases (IDs) are a set of rare diseases that mainly affect growth and metabolism.^1^ The phenomenon of parental imprinting results in the monoallelic expression of a gene, depending on its parental origin.^2^ One of the most studied mechanisms controlling such selective expression is the differential methylation of the imprinting control region (ICR). The region located in chromosome 11p15 is of particular interest, as it contains two ICRs and defects in this region can cause two IDs: Silver-Russell syndrome (SRS, MIM#180860) and Beckwith-Wiedemann syndrome (BWS, MIM#130650). Patients with SRS present with intra-uterine growth retardation, with no catch-up growth after birth, relative macrocephaly at birth, severe feeding difficulties, a protruding forehead, and body asymmetry.^3–5^ The main molecular anomaly identified in these patients is the loss of methylation (LOM) at the *H19/IGF2*:IG-DMR (ICR1) of the paternal allele, resulting in a loss of expression of *insulin-like growth factor 2* (*IGF2*, normally expressed only from the paternal allele during fetal life), a growth factor known to be essential for growth, mainly in the prenatal period (Fig. 1).^6,7^ BWS patients, instead, are usually born with macrosomia, macroglossia, body asymmetry, and an increased risk of developing embryonic tumors in childhood.^8,9^ BWS is either due to a gain of methylation on the maternal ICR1, resulting in overexpression of *IGF2* from the maternal allele, or a LOM of the maternal *KCNQ1OT1*:TSS-DMR (ICR2), resulting in the lack of *cyclin D kinase inhibitor 1c* (*CDKN1c*) expression from the maternal allele. This gene is involved in cycle-cell inhibition (and growth restriction) (Fig. 1).^7^ Patients with IDs present overlapping clinical features that can be explained by the co-regulation of imprinted genes that belong to a dynamic imprinted gene network.^10,11^ Indeed, previous studies have shown that hypomethylation at one locus can affect the expression of maternally or paternally expressed genes of other imprinting loci without affecting methylation of the corresponding ICR.^11–14^ Moreover, Whipple *et al*. recently reported that a cluster of maternally expressed miRNAs can downregulate several imprinted genes expressed from the paternal genome in neurons.^15^ However, one of the limitations in studying these diseases is the low level of expression of imprinted genes in human tissues available for biological research, such as leukocytes or fibroblasts. Hence, several groups have attempted to develop cellular models to explore the mechanisms underlying the pathophysiology of these diseases in tissues involved in the phenotype, such as bone, adipose tissue, hepatocytes, and chondrocytes. Induced pluripotent stem cells, although promising in theory, have shown a general ICR hypermethylation profile.^16–21^ Multipotent stem cells have also been studied recently as an alternative.^22–25^ In this context, it has been shown that mesenchymal stem cells derived from the dental pulp (DPSCs) may be a useful model to study IDs. In a study comparing methylation patterns of DPSCs derived from children with a chromosome 15q11.2-q13.3 maternal duplication (Dup15q), known to be involved in Prader-Willi syndrome (PWS, MIM#176270), to controls, the authors used whole genome bisulfite sequencing to show that methylation markers of 15q11.2-q13.3 duplication were maintained in DPSCs, with significant hypermethylation of the PWS-ICR relative to control DPSCs.^26^ However, the authors did not provide any data about the methylation of other ICRs.^26^ Furthermore, in this last study or in the other published works on cellular models, there is no characterization of the mono- or biallelic expression of imprinted genes in controls or patients with IDs, although this is critical to determine whether parental imprinting is present. Even if this pattern of expression is difficult to assess, the definition of parental imprinting relies on the monoallelic expression of these genes, which makes this analysis critical in IDs’ studies.

To further investigate the potential of DPSCs as a relevant cellular model to study the pathophysiology of IDs, we aimed to (1) characterize the methylation profile of ICRs involved in human IDs in DPSCs of control individuals, (2) assess whether such a methylation profile is modified in patients with ICR methylation defects responsible for their ID, (3) define the expression profile of imprinted genes in these regions and (4) evaluate the dynamics of both the methylation and expression profile in these regions during DPSC osteogenic differentiation.

## Results

### Subjects and cell culture

DPSCs were harvested from five controls (called C1 to C5), with C2 and C3 being extracted from deciduous teeth and C1, C4, and C5 from permanent third molars. We also collected DPSCs from four patients, three with SRS (called SRS1 to 3) and one with BWS (called BWS1). All patient DPSCs were extracted from permanent third molars except for one odontoma (SRS1), with a macroscopic aspect of a deciduous incisor. Clinical follow-up of all patients was at the Hôpital Trousseau Pediatric Endocrinology Clinical Unit, their main clinical characteristics are detailed in Table 1. The molecular diagnosis of SRS was LOM at 11p15 ICR1 for the three patients and BWS was secondary to a LOM at 11p15 ICR2 (Fig. 1).

**Table 1:** Main clinical features of patients with Silver-Russell syndrome (SRS1-3) and Beckwith-Wiedemann syndrome (BWS1)

Items		SRS1	SRS2	SRS3	BWS1
Target height/cm (SDS)		176.0 (0.2)	165.5 (0.45)	182.0 (1.2)	167.0 (0.7)
Birth parameters	Term/(WA + d)	31 + 5	37 + 5	40	33 + 5
	Weight/g (SDS)	890 (−3.5)	1 435 (−4.0)	2 560 (−2.0)	2 700 (2.6)
	Length/cm (SDS)	30.5 (−6.8)	40.5 (−4.7)	45.0 (−3.3)	48.5 (2.5)
	Head circumference/ cm (SDS)	29.0 (−0.1)	32.0 (−1.7)	35.0 (−0.1)	31.5 (0.5)
rGH treatment onset	Age/years	4.0	2.9	2.3	No rGH
	Height/cm (SDS)	87.0 (−3.7)	80.0 (−3.5)	81.7 (−2.0)	No rGH
Last visit	Age/years	13.0	17.5	16.1	20.0
	Height/cm (SDS)	151.5 (−0.1)	150.0^1^ (−2.4)	179.0^1^ (0.7)	179.5^1^ (2.9)
Other features		Body asymmetry, feeding difficulties, advanced puberty	Body asymmetry, severe feeding difficulties with long term nutritional support, advanced puberty	Body asymmetry, feeding difficulties, advanced puberty	Body asymmetry, exomphalos, macroglossia No tumor

*SDS* Standard deviation score, *WA* *+* *d* weeks of amenorrhea and days, *rGH* recombinant growth hormone. ^1^ Final height

**Fig. 2:**
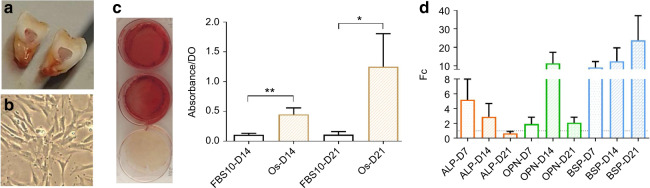
Dental pulp stem cell (DPSC) culture and osteogenic differentiation. **a** Macroscopic view of dental pulp after permanent third molar section. **b** Microscopic image of DPSCs during the cell culture stage (4X/0.10). **c** Red Alizarin staining: the photo on the left shows a macroscopic view of the wells after staining. The two upper wells were cultured in osteogenic medium for 21 days, whereas the lower one was cultured in classical cell culture medium and 10% fetal bovine serum (FBS10). The chart shows the quantification of staining at days 14 and 21 (D14, D21) of cells grown in either osteogenic differentiation (Os, in orange) or classical proliferation medium for the controls and patients (FBS10). OD: optic density. **d** Evolution of expression of *ALP*, *OPN*, and *BSP* within days of differentiation for all individuals. Histograms show the mean value with the standard error of the mean (**P = 0.008, *P = 0.03)

We confirmed the presence of DPSC markers (CD73, CD105, and CD90) in all seeded samples (Supplementary Fig. SD1). The macroscopic and microscopic aspects of the dental pulp and DPSCs in culture are presented in Fig. 2a, b. There were no differences in the morphological aspect of the cells or a delay in proliferation during DPSC proliferation or osteogenic differentiation in vitro. The efficiency of osteogenic differentiation is depicted in Fig. 2c, d, showing both the evolution of expression of the *alkaline phosphatase* (*ALP)*, *osteopontin* (*OPN)*, and *bone sialoprotein* (*BSP*) genes within days of differentiation and Red Alizarin staining. *ALP* expression rose rapidly until day 7 and declined during the last days of osteogenic differentiation, whereas *OPN* expression rose the week after (day 14) and was lower at day 21. Finally, *BSP* expression was high during the entire differentiation process, showing an increase week after week. There was no statistical difference between controls and patients at either day 14 (*P* = 0.49) or day 21 (*P* = 0.8) in Red Alizarin absorbance (Supplementary Fig. SD2a.). There were also no statistical differences in *ALP, OPN*, or *BSP* expression at days 7, 14, or 21 between controls and patients (p from 0.3 to 1.0) (Supplementary Fig. SD2b).

### Methylation of ICRs

**Fig. 3:**
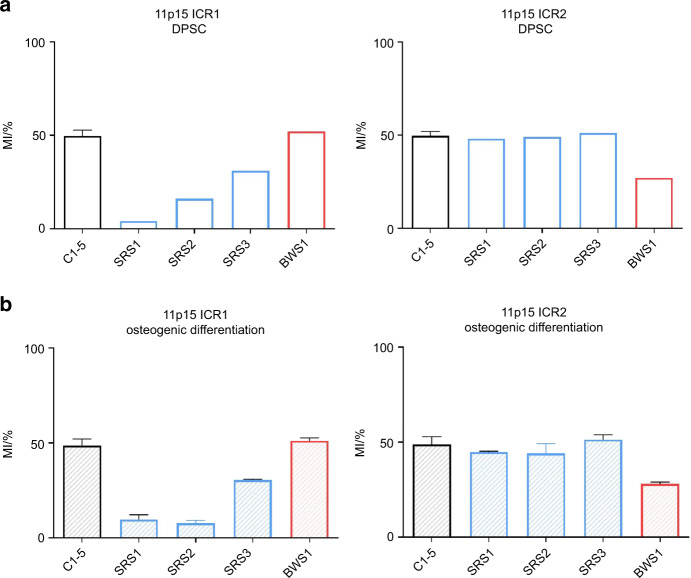
Methylation levels (mean with SD bars) of five controls (C1-5) and four patients (SRS1-3, BWS1) of 11p15 *H19/IGF2*:IG-DMR (ICR1) and *KCNQ1OT1*:TSS-DMR (ICR2). **a** Methylation index (MI) of DPSCs before osteogenic differentiation. MI were determined in a unique experiment for all samples and controls results were pooled. **b** Methylation indices of ICR1 and ICR2 during osteogenic differentiation (days 7, 14, and 21 are pooled, histograms represent the mean and errors bars the standard deviation). Gray zones indicate the normal range of methylation indices identified in controls

**Fig. 4:**
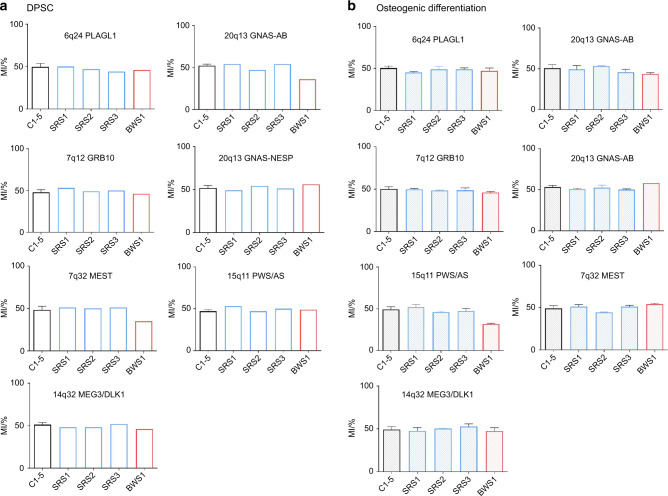
Methylation profile (mean with SD bars) of five controls (C1-5) and four patients (SRS1-3, BWS1) at seven imprinted loci (differentially methylated regions in chromosomes 6q24, 7p12 7q32, 14q32, 15q11, and 20q13). **a** Methylation indices (MIs) of DPSCs. MI were determined with a unique experiment for all samples and controls results were pooled. **b** Methylation indices during osteogenic differentiation (days 7, 14, and 21 are pooled, histograms represent the mean and errors bars the standard deviation). Gray zones indicate the normal range of methylation indices

We studied methylation levels at nine differentially methylated regions (DMRs) loci in five DPSC controls, three SRS patients, and one BWS patient. Methylation levels were normal for all controls at ICRs of 11p15 region (Fig. 3 and Table SD2 in Supplementary Data). As expected, *H19/IGF2*:IG-DMR (ICR1) was hypomethylated for all SRS patients, and *KCNQ1OT1*:TSS-DMR (ICR2) hypomethylated for the BWS patient (Fig. 3). Methylation levels at ICRs of 11p15 region remained unchanged during osteogenic differentiation (Figs. 3 and 4b). The methylation index of 11p15 ICR1 and ICR2 did not vary during several passages (1 to 4) of DPSCs in in-vitro culture (Figure SD3 in Supplementary Data). All seven other DMRs showed normal methylation levels in controls, SRS and BWS patients (Fig. 4 and Table SD2 in Supplementary Data), except for DMR 7q32 and 20q13 of the BWS patient, who had a multi-loci methylation defect both in leukocytes and DPSCs. As in the controls, the methylation levels were stable in SRS and BWS patients during osteogenic differentiation in all studied loci (Fig. 4b).

### Imprinted gene expression

#### Allelic expression

**Fig. 5:**
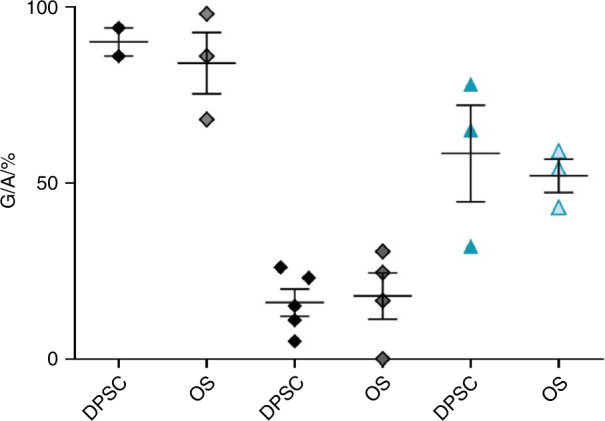
Allelic expression profile of *H19* in individuals with normal methylation at *H19/IGF2*:IG-DMR and one patient with Silver-Russell syndrome (SRS2) in dental pulp stem cells (DPSC) or during osteogenic differentiation (OS). The ratio of each expressed allele was quantified. Values approaching 0 or 100% reflect monoallelic expression, whereas ratios around 50% reflect biallelic expression of *H19*. Each diamond represents one control result (*n* = 3) and each triangle the SRS2 values. Horizontal bars represent the mean of the data and vertical bars the standard error of the mean. rs217727 variation in gDNA: AACCGTCC[A/G]CCGCA

We next determined whether the expression of imprinted genes (mono or biallelic) in this region was concordant with the methylation profiles at ICR1 in both controls and patients (Fig. 1). We thus first searched for polymorphisms in imprinted genes in this region for all individuals (*H19*, *IGF2*). We identified rs217727 (AACCGTCC[A/G]CCGCA) in *H19* exon 5 in two controls (C2 and C4), BWS1, and SRS2. The heterozygous status in this specific genomic site allowed us to determine whether one transcript was predominant (monoallelic expression) or whether both alleles were equally transcribed in the DPSCs. The results are expressed as the ratio of the two transcripts (G/A) (Fig. 5). We found a monoallelic expression profile for both controls and BWS1 in DPSCs under regular culture conditions and during their osteogenic differentiation, in concordance with the methylation indices of these individuals at ICR1. On the contrary, the *H19* expression profile in SRS2 was biallelic in DPSCs and during their osteogenic differentiation, as expected (Fig. 5).

## Discussion

Our results show, for the first time, that all imprinted regions involved in human diseases have a balanced methylation in DPSCs of controls. Our precise quantitative methylation-specific PCR analysis allows us to measure methylation indices similar to those found in the mature cells of other tissues. Furthermore, we confirm that patients already identified to be carrying an abnormal methylation profile at two key ICRs in their leukocytes carry the same methylation defect in their DPSCs. These results are consistent with the only published study from Dunaway et *al*., in which the authors identified a balanced methylation profile in the 15q11 region in controls and an abnormal profile in patients, but no other ICR was investigated.^26^ As imprinting diseases (IDs) represent a very small number of patients, the collection of DPSCs and their analysis for three patients with Silver-Russell syndrome (SRS) and one with Beckwith-Wiedemann syndrome (BWS) is highly valuable.

Osteogenic differentiation of DPSCs resulted in no alterations of the methylation profile for either controls or patients. Furthermore, we did not identify any difference in the efficiency of osteogenic differentiation between controls, SRS, or BWS patients. These data, concordant with those of the study of Dunaway *et al*. on 15q11, confirm the lack of an effect of this differentiation process in the seven ICR methylation profiles that we studied.^26^ Interestingly, Dunaway *et al*. described difficulties in maintaining the stability of the methylome in DPSC cultures, with global hypomethylation and variable methylation profiles between individual DPSC lines for both control and Dup15q. The difficulty to maintain normal levels of methylation in ICRs is also a pitfall in other cellular models, such as induced pluripotent stem cells.^16,27^ Indeed, as imprinted genes work in a co-regulated network, abnormal methylation at one locus can potentially affect the gene expression at other imprinted loci with respect to the methylation of the corresponding ICR.^12–15^ This phenomenon is likely responsible, at least partially, for the clinical overlap identified between several IDs.^11,28–30^ The novelty and strength of our approach is the methylation analysis of all ICRs involved in human IDs. The normal methylation profile in all ICRs we characterized, both in DPSCs and cells generated following osteogenic differentiation, allow their use as a cellular model to study mechanisms underlying the physiopathology of IDs. We also showed that this methylation profile was not altered with time in culture.

On an other hand, Horii *et al*. recently produced an animal model of SRS using epigenome-editing.^31^ They used the dCas9-SunTag and single-chain variable fragment-TET1 catalytic domain to produce an epigenome-edited mouse with ICR1 hypomethylation, upregulation of *H19*, and downregulation of *Igf2* expression relative to control mice. The epigenome-edited mice had SRS phenotype features and additional symptoms out of the SRS clinical spectrum such as cardiac fibrosis probably due to off-target demethylations. Most published studies on the cellular modeling of imprinting diseases only considered the methylation profile at ICRs as a validation of imprint maintenance in the cells, but very few assessed allelic expression to prove it. However, such analysis is difficult due to the small number of polymorphisms reported in the imprinted genes of interest and the existence of mosaicism in the population of cells studied. Indeed, only one group analyzed allelic expression in DPSCs, with the purpose of identifying molecular markers predictive of the efficiency of osteogenic differentiation.^32^ Fanganiello et al*.* performed global gene expression profiling, in which they showed the upregulation of *IGF2* expression in DPSCs relative to that in mesenchymal stem cells from human adipose tissue due to the biallelic expression of *IGF2* in DPSCs.^32^ We urge other groups working on imprinting genes to assess allelic expression of imprinted genes of interest, as it defines imprinting phenomenon and should be a prerequisite to pathophysiological studies. Although limited to three controls and one SRS patient, we demonstrate, using an original method to assess and quantify allelic expression, the preserved correlation between *H19/IGF2*:IG-DMR methylation and allelic expression, confirming maintenance of the imprinting phenomenon. These results show DPSCs to be a potentially relevant model to study parental imprinting.

To conclude, this is the first study in DPSCs obtained from controls and patients with IDs affecting the 11p15 region to characterize both the methylation profile of a large group of ICRs and allelic expression. Our data show that DPSCs and their osteogenic differentiation are a relevant model to study the pathophysiology of Silver-Russell and Beckwith-Wiedemann syndromes. The normal methylation profile observed for the main ICRs inclines us to predict that it could also be a useful cellular model to study other IDs. These results also provide perspectives on DPSC differentiation into other lineages of interest for ID pathophysiology studies.

## Materials and methods

### Cell culture

#### Dental pulp collection

Teeth were obtained after extraction according to an orthodontic treatment plan from the Dental Department of Hôpitaux Universitaires Paris Nord, Université de Paris, Assistance Publique-Hôpitaux de Paris (AP-HP), France. All teeth were collected with informed oral consent from the patients and the parents according to ethical guidelines set by the French law under the approval number 16-024 from the Institutional Review Board -IRB 00006477- of HUPNVS, Paris 7 University and AP-HP in 2016. The origin of the teeth was then coded as C1-5 for controls, SRS1-3 for patients with SRS, and BWS1 for the patient with BWS.

#### DPSC culture

Cultures of human DPSCs were established as previously reported.^22,33,34^ After decontamination with povidone-iodine solution (Betadine, Meda Pharma, France), teeth were longitudinally sectioned and the pulp tissue collected. The pulp was then enzymatically digested with type I collagenase (3 mg·mL^−1^ Worthington Biochem, USA) and dispase (4 mg·mL^−1^ Boehringer Mannheim, Germany). Cells were then seeded in flasks and the cultures maintained with Dulbecco’s Modified Eagle Medium 1 g·L^−1^ d-Glucose (DMEM; Invitrogen, USA) supplemented with 20% fetal bovine serum (FBS; Invitrogen, USA) and 1% penicillin/streptomycin (PS; Invitrogen, USA) at 37 °C with 5% CO_2_. The medium was refreshed twice a week. We did not use specific cell proliferation assay but we monitored the number of days needed between each passage and the delay between first culture and osteogenic differentiation onset for each sample. Cells were detached by trypsinization at 80%-90% confluence (0.25% trypsin EDTA solution Sigma-Aldrich, USA) and either re-plated for proliferation, frozen in liquid nitrogen, or seeded in six-well plates (100 000 cells/well) for osteogenic differentiation. Each step of trypsinization counted as a passage of DPSCs. For all differentiation experiments, DPSCs were used at passages 1 or 2.

#### Osteogenic differentiation

For osteogenic differentiation, DPSCs were seeded in six-well plates and cultured for 21 days with osteogenic medium, composed of DMEM media supplemented with 50 μg·mL^−1^ ascorbic acid sodium salt (Sigma Aldrich, USA), 10^−8^mol·L^−1^ dexamethasone (Sigma Aldrich, USA), 10 mol·L^−1^ β-glycerophosphate (Sigma Aldrich, USA), 10% fetal bovine serum, and 1% penicillin/streptomycin.^35–37^ Medium was renewed twice a week.

#### Staining

At days 14 and 21, cells were fixed in 4% paraformaldehyde for 10 min and Alizarin Red was used as a marker of mineralization. For the quantification of Alizarin Red staining, a 10% acetic acid solution was added to the wells, the plates incubated for 30 min and each cell suspension transferred to a 1.5-mL microcentrifuge tube, heated to 85 °C for 10 min, and centrifuged. The supernatant was transferred to a new tube and the acid neutralized by the addition of 0.4 volumes of 10% ammonium hydroxide. Aliquots were deposited in triplicate in a 96-well plate and absorbance read at 550 nm with a microplate reader (BioTek instruments, Winooski, Vermont, USA).^37,38^

### DNA methylation analysis

#### DNA extraction

DNA was extracted from DPSC cultures before seeding in six-well plates and at days 7, 14, and 21 of osteogenic differentiation. We used an in-house protocol for DNA extraction after cell lysis by a salting-out procedure, as previously described.^11,39^

#### Bisulfite treatment of DNA

Sodium bisulfite treatment of DNA converts all unmethylated cytosine residues to uracil residues. The methylated cytosine residues are unaffected. This process thus generates C/T polymorphisms, which can be used to distinguish between the methylated and unmethylated allele. Genomic DNA (400 ng) was treated with sodium bisulfite using the EZ DNA Methylation lighting kit (Zymo Research, USA), according to the manufacturer’s instructions. Genomic DNA was eluted using 40 μL RNase-free H_2_O and conserved at -20 °C.

#### TaqMan allele-specific methylated multiplex real-time quantitative PCR (ASMM RTQ-PCR) and methylation analysis

The methylation status of seven imprinted loci (nine differentially methylated regions, DMRs) were assessed by ASMM RTQ-PCR, as previously described:^28^ 11p15 *H19/IGF2*:IG-DMR, 11p15 *KCNQ1OT1*:TSS-DMR, 14q32 *MEG3/DLK1*:IG-DMR, 7q32 *MEST* promoter DMR, 7q12 *GRB10*:DMR, 15q11 *PWS/AS*:DMR, 6q24 *PLAGL1*:alt-TSS-DMR, 20q13 *GNAS:*A/B:TSS-DMR, and the *GNAS* locus NESP-DMR. The normal range of methylation indices of the nine DMRs were assessed in five control individuals. The methylation index (MI) at each locus was assigned by calculating the ratio between the methylated and unmethylated alleles as follows: (amount of methylated allele/sum of both methylated and unmethylated alleles) x 100. The ASMM RTQ-PCR primers and probe sequences are provided in supplementary data (Table SD1).

### RNA expression

#### RNA extraction and reverse transcription

RNA was extracted from DPSC cultures before seeding in six-well plates and at days 7, 14, and 21 of osteogenic differentiation. Total RNA was extracted using the NucleoSpin miRNA Kit for the isolation of small and large RNAs (Macherey-Nagel, France). Both DNA and RNA were quantified using a DS-11 spectrophotometer (DeNovix, USA). cDNA was synthesized from long RNA using the miScript PCR System (Qiagen, France) and used for quantitative PCR.

#### Real-time PCR quantification of mRNA

Expression in the controls was arbitrarily set to 1 and fold changes (FCs) between two groups were calculated as FC = 2^−ΔΔCt^. The housekeeping gene *18* *S* was used as a reference because it had the best stability in DPSCs after preliminary experiments comparing it to other commonly used genes. The sequences of all the primers used for PCR are presented in Table SD1 in Supplementary Data.

#### PCR

Qualitative expression of the DPSC-specific markers CD90, CD105, and CD73 was performed for all samples using AmpliTaq Gold 360 Master Mix and a Veriti 96-well thermal cycler (Applied Biosystems, ThermoFisher Scientific, USA). Primer sequences and the amplicon sizes are available in Table SD1 in Supplementary Data.

#### Allelic expression

To assess the mono- or biallelic expression of imprinted genes, we adapted a SNP genotyping assay to cDNA (TaqMan® SNP Genotyping Assays rs217727 C_2603707_10, ThermoFisher Scientific, USA) using TaqPath™ ProAmp™ Master Mix.^40^ First, we searched for rs217727 in *H19* in gDNA of both controls and patients by standard Sanger sequencing (Eurofin Genomics, Germany). The sequencing products were then analyzed using Chromas 2.6.6 (Technelysium Pty Ltd, Australia). Then, we used two probes, one specific for the SNP (rs217727, AACCGTCC[A/G]CCGCA) and the other for DNA with no polymorphism. Finally, we quantified the relative expression of cDNA (reversed from mRNA) carrying the polymorphism or not by RTQ-PCR. Results are expressed as the ratio of cDNA carrying a G or A in the rs217727 position. Primer and probe sequences are provided in Supplementary Data (Table SD1).

#### Statistics

Data presented in the figures are expressed as means (for controls and SRS patients) and the error bars represent the standard error of the mean (SEM). Mann-Whitney tests were used to compare unpaired quantitative distributions and Wilcoxon tests if the values were paired. All graphs were created and statistics performed using GraphPad Prism 6 (USA).

## Supplementary information

Supplemental data
