# Supplementary material for: Dental pulp stem cells as a promising model to study imprinting diseases
Source: Int J Oral Sci. 2022 Apr 2;14:19. doi: 10.1038/s41368-022-00169-1 (PMC8976849; doi:10.1038/s41368-022-00169-1)
Supplement: Supplementary file 1 — Supplemental data [file 41368_2022_169_MOESM1_ESM.docx]

**Dental pulp stem cells as a promising model to study imprinting diseases**

Eloïse Giabicani, Aurélie Pham, Céline Sélénou, Marie-Laure Sobrier, Caroline Andrique, Julie Lesieur, Agnès Linglart, Anne Poliard, Catherine Chaussain, and Irène Netchine.

# Supplementary data

**Table SD1.** List and sequences of the primers used in this study. F: forward, R: reverse, M: methylated allele, UM: unmethylated allele

| **Primers used for mRNA amplification by PCR** | | | |
| --- | --- | --- | --- |
| **Primer** | **5’ Sequence 3’** | | **Amplicon size** |
| CD105-F | AGTCTTGCAGAAACAGTCCA | | 129bp |
| CD105-R | TGGACTTCAAGGATGGCATT | |  |
| CD90-F | AATACCAGCAGTTCACCCAT | | 165bp |
| CD90-R | GCTAGTGAAGGCGGATAAGT | |  |
| CD73-F | AGCTTACGATTTTGCACACC | | 130bp |
| CD73-R | ATCTGCTGAACCTTGGTGAA | |  |
| **Primers used for gDNA and cDNA amplification and sequencing** | | | |
| **Primer** | | **5’ Sequence 3’** | |
| H19-ex1F | | CAGTCACCCGGCCCAGAT | |
| H19-ex1R | | AAGACACCATCGGAACAGCA | |
| H19-ex4-5R | | GCTCTGGAAGGTGAAGCTAG | |

| **Primers used for mRNA quantification by RT-PCR** | |
| --- | --- |
| **Primer** | **5’ Sequence 3’** |
| IGF2-F | CGGCTTCCAGACACCAATGG |
| IGF2-R | GCGGAAACAGCACTCCTCAA |
| COL1A1-F | TGACTGGAAGAGTGGAGAGTA |
| COL1A1-R | TCTTGCTGATGTACCAGTTCT |
| OPN-F | GTTGTCCCCACAGTAGACAC |
| OPN-R | GTATGCACCATTCAACTCCT |
| ALP-F | AACTGATGTGGAGTATGAGAGTG |
| ALP-R | GAAGTGGGAGTGCTTGTATCT |

| **Primers and probes used for TaqMan allele-specific methylated multiplex real-time quantitative PCR (ASMM RTQ-PCR)** | | |
| --- | --- | --- |
| **Locus** | **Primers** | **Probes** |
| 11p15  *H19/IGF2*:IG-DMR | F : GTTTTTATATTATAGTTTAAGTTYGTTTTAGTTGAGGT | M : ACGTAACCCGAAACGT |
|  | R : TCCCATAAATATTCTATCCCTCACTACC | UM : CTTACATAACCCAAAACATT |
| 11p15  *KCNQ1OT1*:TSS-DMR | F : GGGTTAGTTTTTTGYGTGATGTGTTT | M : ATAGCGGTCGTATTTCGATAT |
|  | R : ACCTCCACACCRAAAACCCA | UM : TGTGTGAGGATAGTGGTTGT |
| 14q32  *MEG3/DLK1*:IG-DMR | F : AGTTTTATGTTAAGATGTTAATTATTTTTTGGA | M : TAGTTCGCGGTTCGTTAT |
|  | R : ACCAAAAAACCTAACAAATCAAAACA | UM : TAGTAGTTTGTGGTTTGTTAT |
| 7q32  *MEST* promoter DMR | F : GTATTAGGGTGAGATTAGGGTTATTATGG | M : TATTACGACGATTTCGG |
|  | R : AAATATCACTCCTACCCRCAATAAAAT | UM : AAATTTTATTATGATGATTTT |
| 7q12  *GRB10*:DMR | F : GGTTGTAATATTTAGTTTTYGAGGGATTG | M : ACGGAGTAGCGTTCGGT |
|  | R : CRTCACATAAATTTAATCCTAAAATTCCTAT | UM : ATGGAGTAGTGTTTGGTTTT |
| 15q11  *PWS/AS*:DMR | F : GATTTTGAGYGGTGTTTTATAGTTTAGGTT | M : AACGCATTTACTACGAAC |
|  | R : TCCTATAACCTTCCCACAACRCAC | UM : TAACACATTTACTACAAACCA |
| 6q24  *PLAGL1*:alt-TSS-DMR | F : AYGGGTTGAATGATAAATGGTAGATGT | M : CCGTTCCGACTCCCGA |
|  | R : AACCCCAACRCCCTCCTC | UM : CACCATTCCAACTCCCAAA |
| 20q13 | F: TGTTTGGGAGAGGATAGAGGAGA | M : AGTTGCGCGTTCGGA |
| *GNAS* locus NESP-DMR | R: ACCCCCACCTCCTTACTACACA | UM :TGTGAAGTTGTGTGTTTGGA |
| 20q13 | F: AGTAGGGGAGGTTTTGTATTTTTATYGTA | M : TCGTAAATAATTCGAATT |
| *GNAS*:A/B:TSS-DMR | R: TCCTTACCTCCAACCACCCAC | UM : TTGGTGGGTGTGTGTGTTGT |

**Figure SD1.** CD90 (**a**), CD105 (**b**), and CD73 (**c**) expression in controls (C1-C5), SRS patients (SRS1-3), and the BWS patient (BWS1) relative to that in fibroblasts (F), as a positive control, and water (H_2_0), as a negative control.


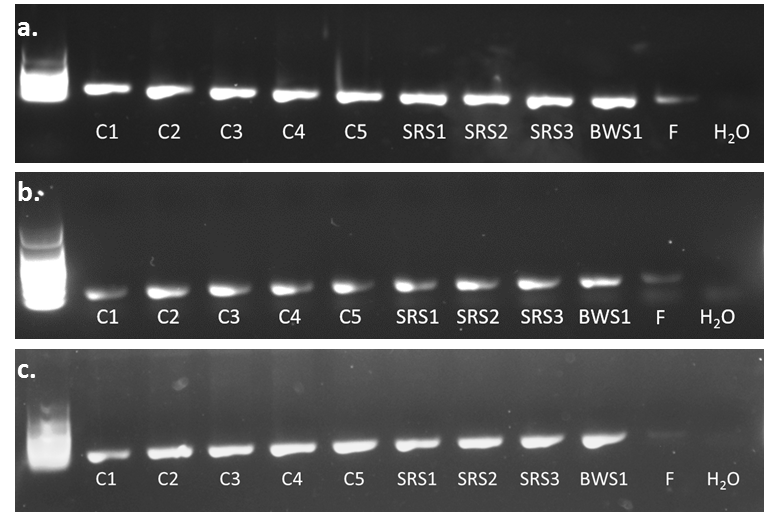


**Figure SD2.** Comparison of osteogenic differentiation characteristics between controls, SRS patients, and the BWS patient. **a.** Quantification of Red Alizarin absorbance in DPSCs cultured in osteogenic medium relative to that of cells not exposed to osteogenic differentiation at days 14 (D14) and 21 (D21) of differentiation. **b, c, and d.** Relative expression of *ALP, OPN,* and *BSP* at days 7, 14, and 21 of osteogenic differentiation. There was no statistical difference between the controls and SRS patients.


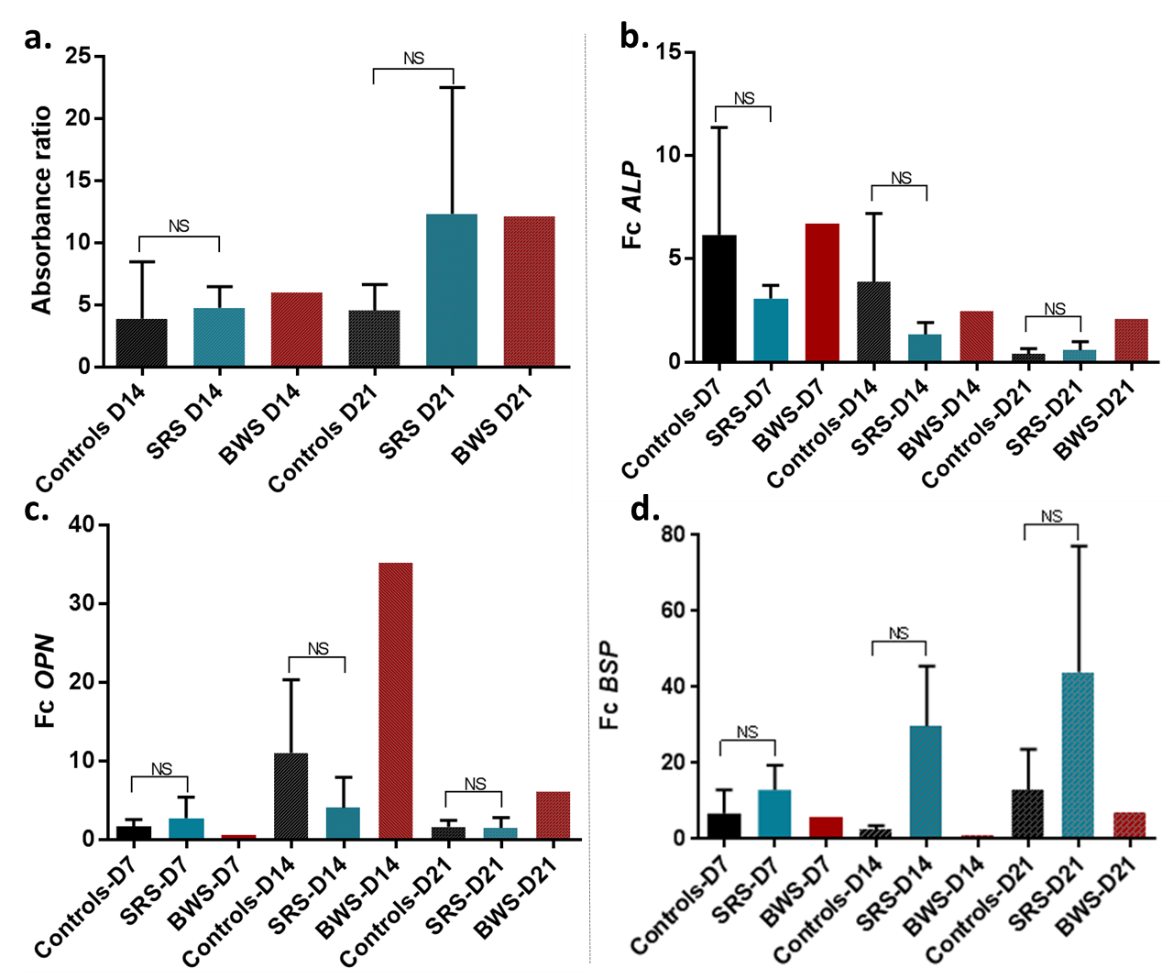


**Figure SD3.** Profile of methylation (mean with SD bars) of **a.** four controls (C1, C2, C4 and C5) and **b.** four patients (SRS1-3, BWS1) at ICR1 and ICR2 at various passages of DPSCs in *in vitro* culture. MI: methylation index.


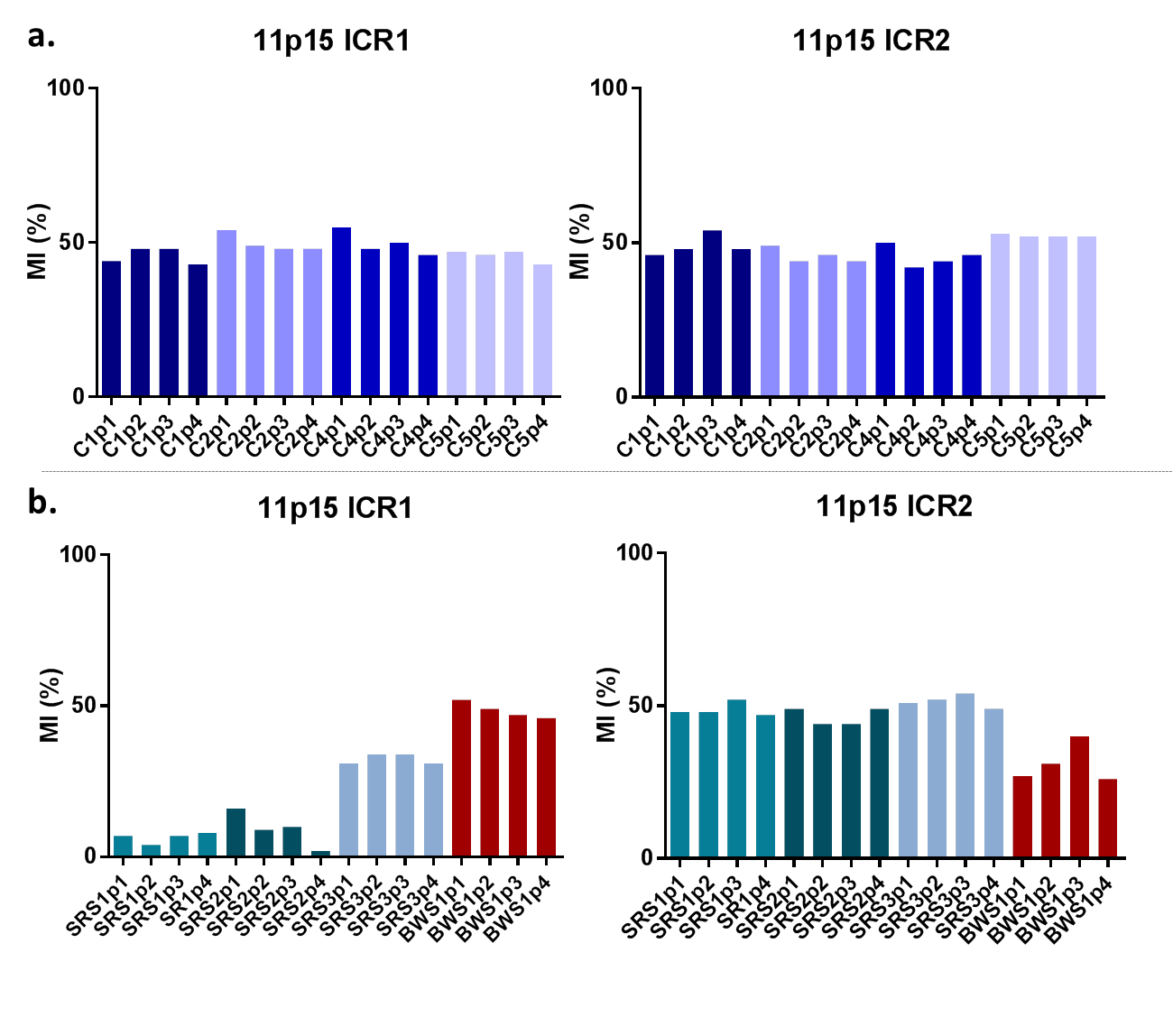


**Table SD2.** Methylation levels for all patients (SRS1-3, BWS1) and controls (C1-5) at various passages during *in vitro* culture of DPSCs and osteogenic differentiation (days 7, 14, and 21), as determined by ASMM RT-qPCR in the 9 DMRs studied. Low levels of methylation are highlighted in green and high levels in orange.

|  | **11p15 ICR1** | **11p15 ICR2** | **14q32**  **DLK1** | **7q32 MEST** | **7p12 GRB10** | **15q11 PWS/AS** | **6q24**  **PLAGL1** | **20q13 GNAS-AB** | **20q13 GNAS-NESP** |
| --- | --- | --- | --- | --- | --- | --- | --- | --- | --- |
| C1 p1 | 44 | 46 | 47 | 51 | 51 | 45 | 53 | 52 | 52 |
| C1 p2 | 48 | 48 | 51 | 45 | 50 | 48 | 53 | 55 | 52 |
| C1 p3 | 48 | 54 | 51 | 50 | 50 | 50 | 50 | 54 | 52 |
| C1 p4 | 43 | 48 | 52 | 47 | 54 | 44 | 54 | 56 | 54 |
| C1 day 7 | 43 | 51 | 52 | 51 | 49 | 47 | 54 | 53 | 52 |
| C1 day 14 | 48 | 53 | 47 | 52 | 51 | 48 | 49 | 56 | 52 |
| C1 day 21 | 48 | 45 | 47 | 45 | 51 | 47 | 52 | 53 | 53 |
| C2 p1 | 54 | 49 | 52 | 43 | 49 | 46 | 45 | 50 | 56 |
| C2 p2 | 49 | 44 | 49 | 46 | 48 | 48 | 49 | 42 | 52 |
| C2 p3 | 48 | 46 | 47 | 51 | 47 | 48 | 46 | 53 | 53 |
| C2 p4 | 48 | 44 | 48 | 50 | 48 | 49 | 45 | 54 | 52 |
| C2 day 7 | 47 | 49 | 47 | 48 | 47 | 47 | 49 | 44 | 53 |
| C2 day 14 | 53 | 56 | 56 | 54 | 57 | 46 | 49 | 44 | 58 |
| C2 day 21 | 45 | 50 | 51 | 53 | 50 | 48 | 49 | 51 | 52 |
| C3 p1 | 52 | 48 | 54 | 47 | 49 | 48 | 48 | 52 | 50 |
| C3 day 7 | 46 | 45 | 49 | 44 | 47 | 49 | 50 | 49 | 53 |
| C3 day 14 | 51 | 47 | 44 | 51 | 48 | 48 | 48 | 44 | 52 |
| C3 day 21 | 51 | 48 | 48 | 54 | 50 | 49 | 48 | 48 | 55 |
| C4 p1 | 55 | 50 | 52 | 48 | 49 | 45 | 51 | 51 | 48 |
| C4 p2 | 48 | 56 | 51 | 43 | 47 | 44 | 47 | 51 | 54 |
| C4 p3 | 50 | 44 | 49 | 48 | 50 | 48 | 50 | 54 | 56 |
| C4 p4 | 46 | 46 | 44 | 45 | 52 | 46 | 51 | 54 | 56 |
| C4 day 7 | 54 | 54 | 52 | 48 | 50 | 45 | 49 | 55 | 55 |
| C4 day 14 | 53 | 57 | 50 | 49 | 50 | 47 | 48 | 57 | 52 |
| C4 day 21 | 50 | 46 | 47 | 46 | 52 | 49 | 51 | 54 | 52 |
| C5 p1 | 47 | 53 | 47 | 54 | 49 | 49 | 55 | 53 | 53 |
| C5 p2 | 46 | 52 | 47 | 51 | 56 | 51 | 50 | 47 | 51 |
| C5 p3 | 47 | 52 | 46 | 46 | 53 | 54 | 51 | 54 | 50 |
| C5 p4 | 43 | 52 | 50 | 46 | 53 | 54 | 48 | 54 | 52 |
| C5 day 7 | 43 | 46 | 45 | 46 | 48 | 54 | 50 | 49 | 53 |
| C5 day 14 | 48 | 46 | 48 | 47 | 48 | 57 | 52 | 52 | 49 |
| C5 day 21 | 49 | 53 | 55 | 51 | 55 | 53 | 55 | 53 | 53 |
| SRS1 p1 | 7 | 48 | 47 | 50 | 52 | 53 | 52 | 47 | 49 |
| SRS1 p2 | 4 | 48 | 48 | 51 | 53 | 53 | 50 | 54 | 49 |
| SRS1 p3 | 7 | 52 | 48 | 52 | 57 | 53 | 50 | 54 | 51 |
| SRS1 p4 | 8 | 47 | 49 | 53 | 50 | 53 | 49 | 51 | 49 |
| SRS1 day 7 | 10 | 45 | 45 | 49 | 49 | 53 | 46 | 53 | 50 |
| SRS1 day 14 | 12 | 44 | 46 | 55 | 51 | 52 | 46 | 44 | 52 |
| SRS1 day 21 | 7 | 45 | 52 | 52 | 49 | 48 | 44 | 51 | 50 |
| SRS2 p1 | 16 | 49 | 48 | 50 | 49 | 47 | 47 | 47 | 54 |
| SRS2 p2 | 9 | 44 | 50 | 47 | 46 | 44 | 48 | 54 | 53 |
| SRS2 p3 | 10 | 44 | 49 | 48 | 50 | 46 | 46 | 50 | 53 |
| SRS2 p4 | 10 | 49 | 48 | 48 | 51 | 43 | 45 | 51 | 52 |
| SRS2 day 7 | 8 | 56 | 50 | 46 | 48 | 44 | 45 | 53 | 56 |
| SRS2 day 14 | 6 | 41 | 51 | 45 | 48 | 45 | 50 | 53 | 50 |
| SRS2 day 21 | 9 | 50 | 50 | 47 | 49 | 44 | 52 | 54 | 51 |
| SRS3 p1 | 31 | 51 | 52 | 51 | 50 | 50 | 44 | 54 | 51 |
| SRS3 p2 | 34 | 52 | 58 | 44 | 53 | 49 | 46 | 44 | 53 |
| SRS3 p3 | 34 | 54 | 53 | 48 | 48 | 48 | 46 | 47 | 51 |
| SRS3 p4 | 31 | 49 | 53 | 48 | 49 | 50 | 45 | 50 | 49 |
| SRS3 day 7 | 30 | 54 | 55 | 45 | 52 | 49 | 51 | 44 | 50 |
| SRS3 day 14 | 30 | 49 | 49 | 46 | 47 | 52 | 48 | 43 | 51 |
| SRS3 day 21 | 31 | 51 | 54 | 51 | 47 | 52 | 48 | 50 | 49 |
| BWS1 p1 | 52 | 27 | 46 | 35 | 46 | 49 | 46 | 36 | 56 |
| BWS1 p2 | 49 | 31 | 46 | 32 | 50 | 48 | 47 | 36 | 61 |
| BWS1 p3 | 47 | 40 | 45 | 33 | 49 | 51 | 49 | 26 | 59 |
| BWS1 p4 | 46 | 26 | 46 | 30 | 48 | 50 | 49 | 31 | 55 |
| BWS1 day 7 | 50 | 27 | 44 | 31 | 46 | 53 | 46 | 42 | 58 |
| BWS1 day 14 | 53 | 28 | 52 | 33 | 45 | 54 | 44 | 44 | 58 |
| BWS1 day 21 | 50 | 29 | 46 | 31 | 47 | 55 | 51 | 45 | 58 |
